# Improving Communication Pathways – A Technological Intervention

**DOI:** 10.1192/bjo.2025.10394

**Published:** 2025-06-20

**Authors:** Mohamed Mazin, Noura Filali, Maryam Ali, Srinivas Gopi, Sonika Bhasin

**Affiliations:** Hertfordshire NHS Trust, London, United Kingdom

## Abstract

**Aims:** To streamline the communication process for doctors by integrating essential contact numbers into the Accurix system, making information readily accessible and reducing time spent searching for contact details.

**Methods:** Pre-intervention survey among junior doctors; Integration of trust directory into Accurix; Post-intervention survey with analysis of results and feedback.

**Results:** Before: Ease of locating correct ward/unit numbers:

58.6% find it difficult (rating 1–2 out of 5).

34.5% neutral (rating 3 out of 5).

Only 6.9% find it easy (rating 4–5 out of 5).

Before: Current methods for finding phone numbers:

65.5% use Google.

62.1% use WhatsApp Group Chat/Word document.

51.7% use KFC Doctor Office.

After: Ease of locating correct ward/unit numbers:

Now, nearly 60% find it easy (rating 4–5 out of 5).

After: Current methods for finding phone numbers:

With 70% of people now using Accurix.

**Conclusion:** The implementation of our technological intervention has significantly transformed the way healthcare professionals communicate across different units, leading to improved patient care and efficiency. Prior to the introduction of the app, many clinicians faced considerable challenges when attempting to contact colleagues in other units. The reliance on outdated methods, such as word of mouth, personal contacts, or searching through Google, often resulted in delays, miscommunication, and frustration. These inefficiencies not only hindered the flow of information but also impacted patient care, as timely communication is critical.

The Accurix app, which serves as a comprehensive and up-to-date phone directory for multiple trusts, has addressed these issues head-on. Since its introduction, the majority of doctors across the trusts have adopted the app, with usage rates steadily increasing. Feedback from users has been overwhelmingly positive, with many reporting that the app has made it significantly easier to contact the right person at the right time. This streamlined communication has had a direct impact on patient care, as doctors can now quickly consult with professionals, arrange transfers, or coordinate care across units without unnecessary delays.

The reduced dependence on informal communication methods, such as word of mouth or searching for contact details online, has not only saved time but also improved the accuracy and reliability of information. This has led to a more efficient patient flow, as clinicians can now focus on delivering care rather than spending valuable time trying to track down contact information. Furthermore, the app has contributed to a more standardized approach to inter-trust communication, ensuring that all healthcare professionals have access to the same resources, regardless of their location or specialty.

